# Angèle van den Ven, Lodewijk Schmit Jongbloed: Arzt & Ärztin als Ganzes – sinnvoll arbeiten, sinnvoll leben

**DOI:** 10.3205/zma001429

**Published:** 2021-02-15

**Authors:** Udo Obertacke, Johannes Obertacke

**Affiliations:** 1 Universitätsmedizin Mannheim, Orthopädisch-Unfallchirurgisches Zentrum, Mannheim, Germany; 2 Universitätsmedizin Mannheim, Chirurgische Klinik, Mannheim, Germany

## Bibliographical details

Angèle van de Ven, Lodewijk Schmit Jongbloed

**Arzt & Ärztin als Ganzes – sinnvoll arbeiten, sinnvoll leben**

Verlag: Schmit Joengbloed Advies Verlag, Oegstgeest (NL)

Year of publication: 2020, pages: 108

## Recension

Book review by Udo Obertacke (father, older physician) and Johannes Obertacke (son, younger physician)

*Arzt & Ärztin als Ganzes *is a short, straightforward book (104 pages, small format, translated into German) by Dutch authors wanting to give medical doctors new suggestions for approaching work and life and to ask probing questions about professional and personal development. They describe different career and life phases.

### Father

Medical doctors have always felt pulled in four different directions: their self-perception (as academics with ideas about their work and its appropriate remuneration), society’s perception (expectations of the best-possible care, 24/7 and free of charge), treatment in the doctor’s immediate surroundings (usually in a hospital that employs him or her in a position subjectively felt to be inferior, at least until the doctor attains recognition as a medical specialist, after which comes the economic pressure – with the same intent), and – last but not least – the reaction of the patients (who cling to the doctor, by whom they feel understood and cared for, and engage in all forms of psychological projection; it is all about the patient and only the patient!) 

It would also be accurate to describe the book as a brief guideline for the young physician (searching for direction?) and also the older physician (facing a loss of motivation?). The book has several chapters but no table of contents, since it is meant to be read from beginning to end (p. 32) and offers many quotes and in part subtle images and comics (alone on 23 of 104 pages).

Right at the beginning, the book details the reflective phase of the young medical student who objectively perceives his or her career as “quite good” but the prospects as “scary.”

The book then goes on to cover the viewpoints of medical interns regarding the choice of a specialty, the assistant physician, the “newly credentialed” specialist (astonishingly, the recommended website, [https://www.themedicportal.com/] (p. 43), recognized the father’s profession and the son’s intended postgraduate goal by asking six innocuous questions), the experienced physician, and the wise doctor. The sequences of possible decisions are presented step by step and completely realistically.

The authors encourage taking detours (in both professional and personal development), share tips on discovering roles (however, the analogy of “actor” goes too far in my opinion; where is the doctor’s authenticity?), call for “space to think” and mindfulness of self, and give advice on burnout (is that really important nowadays or is it Zeitgeist?).

Some explanations and recommendations touch on wellness esotericism (team meetings, advising by the human resource department, personal surprises, etc.), because where real problems are identified (choosing a specialty, motivation), the proposed solutions are in part too abstract or just plain shallow. On the other hand, very real information and figures are given (percentage of (dis)satisfied assistant physician trainees, number of career changers, post-licensure education credits, etc.).

The older physician now proceeds to form some thoughts on the authors: A figure (p. 82) shows a correlation between professional development (y-coordinate) and age (x-coordinate); the highest point on the curve is achieved in the medical specialist phase. This is not true for most of the professional medical careers I have witnessed and this idea may irritate young colleagues also.

Such books are important and brevity is the soul of wit (acceptance). No one would read a work that attempted to explain the entire sociology and psychology of the medical profession (and perhaps no one would believe it either). A little guidebook and troublemaker for 14.95€.

#### Son

To my understanding, this book intends to show the reader that each phase of the medical career has different priorities, goals, desires and problems. Roughly summarized, it aims to encourage awareness of these particular aspects and adjustment to them as necessary. I wish to back this stance strongly, because precisely these are important topics for doctors: workload, gratification crises, and doubts about whether the chosen path is the right one. Not the least because medical doctors spend a significant part of their lives working and hope, in the best of cases, to be happy in that work.

I am not aware of any career counseling offered by employers in Germany.

I think that the degree to which professionalism is achieved over the course of a medical career can vary incredibly widely among physicians. The curve appearing on page 82 seems questionable to me and that it would at least provoke those who discuss and research the topic of life-long learning.

## Competing interests

The authors declare that they have no competing interests. 

